# Metabolic Rewiring and Post-Translational Modifications: Unlocking the Mechanisms of Bone Turnover in Osteoporosis

**DOI:** 10.14336/AD.2025.0123

**Published:** 2025-03-24

**Authors:** Yuanyuan Li, Qilin Li, Kehan Zhang, Yaxin Wu, Gaoshaer Nuerlan, Xiangyao Wang, Yuxiao Zhang, Ahsawle Ozathaley, Jing Mao, Yan Liu, Shiqiang Gong

**Affiliations:** ^1^Center of Stomatology, Tongji Hospital, Tongji Medical College, Huazhong University of Science and Technology, Wuhan, China.; ^2^School of Stomatology, Tongji Medical College, Huazhong University of Science and Technology & Hubei Province Key Laboratory of Oral and Maxillofacial Development and Regeneration, Wuhan, China.; ^3^Laboratory of Biomimetic Nanomaterials, Department of Orthodontics & National Center for Stomatology & National Clinical Research Center for Oral Diseases & National Engineering Laboratory for Digital and Material Technology of Stomatology & Beijing Key Laboratory of Digital Stomatology & Research Center of Engineering and Technology for Computerized Dentistry Ministry of Health & NMPA Key Laboratory for Dental Materials & Translational Research Center for Orocraniofacial Stem Cells and Systemic Health, Peking University School and Hospital for Stomatology, Beijing 100081, China.

**Keywords:** osteoporosis, metabolites, post-translational modifications, osteoblast differentiation, osteoclast differentiation

## Abstract

Osteoporosis is a metabolic disease characterized by low bone density resulting from abnormal bone metabolism, caused by impaired osteogenesis and/or excessive bone resorption. The coordinated differentiation of osteoblasts (originating from mesenchymal stem cells) and osteoclasts (derived from hematopoietic progenitor cells) is necessary for maintaining normal bone remodeling and homeostasis. Metabolites have been confirmed to regulate cellular behavior through post-translational modifications (PTMs), including acetylation, lactylation, and succinylation. During osteoblast and osteoclast differentiation, progenitor cells undergo metabolic rewiring to meet the energy demands of these biological processes. Consequently, local metabolite profiles and intermediate metabolic products dynamically change during bone remodeling, influencing cell differentiation via PTMs. Given the regulatory role of PTMs in bone metabolism, this review systematically examines PTMs involved in osteoblast and osteoclast differentiation and explores potential avenues for addressing osteoporosis.

## 1. Introduction

Aging is a natural process characterized by the gradual decline of an organism's physiological functions, influenced by the combined effects of multiple factors. With aging, the prevalence of serious diseases increases, including neurodegenerative disorders, cardiovascular diseases, and skeletal diseases [1]. Among these, osteoporosis is a typical age-related bone disease characterized by deterioration of bone microstructure and reduced bone mineral density, significantly increasing fracture risk [2, 3]. Genetic variants and hormonal imbalances also account for osteoporosis. Among hormones, estrogen deficiency is the major cause of postmenopausal osteoporosis. Affecting millions of aging individuals annually, osteoporosis has emerged as a pressing public health concern [4, 5]. However, the unclear etiology and pathogenesis of osteoporosis hinder progress in clinical management. Therefore, elucidating the pathological mechanisms underlying osteoporosis is the prerequisite for the treatment of osteoporosis. Bone is a dynamic organ that undergoes continuous remodeling throughout life. Under physiological conditions, osteoblasts secrete bone matrix and facilitate its mineralization to maintain new bone formation, whereas osteoclasts secrete acidic substances and proteases to mediate bone resorption. Abnormal bone turnover, which is characterized by excessive osteoclast activity and insufficient osteoblast differentiation, is the primary driver of osteoporosis [6, 7].

Cell metabolism plays a central role in producing adenosine triphosphate (ATP), the main energy source for biological activities. Beyond ATP, metabolites generated from cell metabolism, including acetyl-CoA, lactate, succinate, palmitic acid, and citrulline, also regulate cellular processes through post-translational modifications (PTMs) [8]. PTMs refer to the addition or removal of small molecules from the amino acid residues in the polypeptide chain of proteins, including histones and non-histones. Thus, PTMs result in conformational changes and functional alterations of proteins. Histone modifications, as a key mechanism of epigenetic regulation, induce alterations in chromatin structure. The structural changes influence interactions between transcription factors and the transcription start sites (TSS) of downstream genes. This regulation ultimately activates or silences gene transcription. Non-histone modifications, on the other hand, regulate protein stability and functionality. In recent years, the roles of PTMs in cancer, degenerative diseases, and inflammation have been extensively studied [9]. However, the role of PTMs in bone metabolism has not been systematically summarized

Metabolic reprogramming occurs during cell differentiation and results in dynamic changes in metabolite profiles. Meanwhile, several metabolites have been demonstrated to regulate osteoblast or osteoclast differentiation and contribute to osteoporosis through PTMs [10]. Thus, PTMs with metabolites as substrates serve as a link between complex cell metabolism and the regulation of cellular functions. Focusing on the role of PTMs utilizing metabolites as substrates in bone remodeling, specifically in osteoblast and osteoclast differentiation, will be instrumental in unraveling the pathology of osteoporosis. In this review, we summarize the latest findings on PTMs that utilize metabolites as substrates and their role in osteoblast and osteoclast differentiation. We aim to provide insights into potential strategies for leveraging PTMs, including histones and non-histones, to promote osteoblast differentiation and/or suppress excessive osteoclast activity, ultimately improving bone mass in patients with osteoporosis.

## 2. Abnormal bone turnover in senile osteoporosis

The skeletal system undergoes dynamic remodeling throughout life, with osteoblasts driving bone formation and osteoclasts facilitating bone resorption [11, 12]. Osteoblasts originate from mesenchymal stem cells (MSCs), especially bone marrow mesenchymal stem cells (BMSCs). Other MSC populations, such as umbilical cord mesenchymal stem cells (UCMSCs), periodontal ligament stem cells (PDLSCs), dental pulp stem cells (DPSCs), adipose stem cells (ADMSCs), and others, reside in different tissues and have the potential to differentiate into osteoblasts under certain stimulation [13]. In contrast, osteoclasts are derived from the hematopoietic cell lineages, particularly the monocyte/macrophage system.

Usually, bone remodeling initiates with osteoclast differentiation from progenitor cells. Under the stimulation of receptor activator of nuclear factor kappa-B ligand (RANKL) and macrophage colony-stimulating factor (M-CSF), monocyte/macrophage-derived cells fuse and form multinucleated osteoclasts to degrade bone matrix [14]. Bone resorption is mainly mediated by proteases, such as matrix metalloproteinases (MMPs) and cathepsin K, secreted by osteoclasts. As osteoclasts mature and degrade the matrix, growth factors such as transforming growth factor-β1 (TGF-β1) and insulin-like growth factor 1 (IGF1) are released from the bone matrix, initiating the reversal phase and subsequent osteogenesis [15, 16]. Therefore, these factors recruit MSCs to differentiate into osteoblasts to achieve bone formation under stimulation. Moreover, extracellular vesicles (EVs) derived from osteoclasts could carry RANK, which integrates with RANKL on the membrane of osteoblast progenitor cells, thereby triggering mammalian target of rapamycin (mTOR) signaling to facilitate osteoblast differentiation [17]. Bone matrix consists of water, organic collagen, and minerals. Osteoblasts can secrete type Ⅰ collagen, which is the main component of organic collagen [18]. The mineralization of the extracellular matrix contributes to the mechanical properties of the bone. Before the onset of bone formation, osteoclasts undergo apoptosis under the stimulation of pro-apoptotic signaling or convert into inactive intermediate cells, such as osteomorphs, to avoid untimely resorption of the newly formed bone [14]. After the refilling of the focal resorption site, osteoblasts subsequently undergo apoptosis or differentiate into bone lining cells and osteocytes [16]. When stimuli occur, bone remodeling recurs to maintain skeletal health by degrading old bone and forming new bone. Obviously, osteoclasts and osteoblasts are the main participants in bone remodeling. The elaborate intercellular communication fine-tunes the coordinated bone remodeling.

However, in osteoporosis, aging-induced changes result in decreased osteogenesis, excessive bone degradation, reduced bone strength, and increased bone fragility. A major feature of aging is the accumulation of senescence-associated secretory phenotype (SASP). SASP is mainly characterized by increased inflammatory secretions, including proinflammatory cytokines, vesicles, and reactive oxygen species (ROS) in the microenvironment. Studies have shown that senescent BMSCs tend to preferentially differentiate into adipocytes rather than osteoblasts, leading to increased bone marrow fat and reduced new bone formation [19-21]. This might be caused by SASP, since SASP induced by ROS has been demonstrated to induce MSC senescence [22]. Although some researchers suggest that osteoporosis primarily results from limited bone formation, other studies demonstrate that SASP could stimulate excessive osteoclast activation, causing severe bone erosion [23, 24]. Apart from the direct impacts on osteoblasts and osteoclasts, SASP could also stimulate the inflammatory phenotype of immune cells, disrupting bone metabolism and inducing osteoporosis [25]. Notably, genetic variants and hormonal imbalances also account for at least part of the pathogenesis of osteoporosis. Single nucleotide polymorphisms (SNPs) are common heritable genetic variations. Notably, SNPs in the genes of estrogen receptor (ER) α and major histocompatibility complex are associated with postmenopausal osteoporosis [26]. Estrogen is regarded as a key bone protective hormone, as it promotes osteogenesis through Wnt signaling and inhibits osteoclastogenesis via upregulation of osteoprotegerin (OPG) [27]. Given its critical role in bone formation, estrogen deficiency is induced by ovariectomy (OVX) and utilized to simulate reduced estrogen levels in postmenopausal women, thereby inducing osteoporosis in female mice or rats [28]. Glucocorticoids (GCs) are also indispensable for skeletal homeostasis. Physiologically, GCs induce the expression of Wnt, bone morphogenetic protein (BMP), and OPG in osteoblasts and osteocytes, thereby facilitating bone formation [29]. Nonetheless, the long-term administration of GCs as medicine results in excessive bone mass reduction, primarily due to impaired osteogenesis and enhanced osteoclastogenesis [29]. Similar to GCs, parathyroid hormone (PTH), secreted by the parathyroid gland, is also essential for skeletal health. The role of PTH in bone remodeling depends on its dose and duration. Intermittent administration of PTH facilitates osteogenesis through PTH type 1 receptor (PTH1R) signaling. By binding to PTH1R, PTH activates protein kinase A (PKA) and protein kinase C (PKC) signaling to enhance the proliferation, recruitment, and differentiation of osteoblasts [30]. However, long-term and continuous utilization of PTH leads to excessive bone resorption. Osteoclasts fail to express PTH1R. Thus, under persistent PTH stimulation, excessive activation of osteoclasts results from elevated RANKL secretion by osteoblasts, osteocytes, and T cells [31]. Despite the complex pathology, osteoporosis is ultimately caused by abnormal bone metabolism, characterized by an imbalance between bone resorption and formation. It is necessary to elucidate the mechanisms of excessive osteoclastogenesis and/or impaired osteogenesis in osteoporosis.

## 3. Metabolic rewiring mediates bone metabolism

Glucose, amino acids, and lipid metabolism are the major metabolic pathways in cells. During osteoblast or osteoclast differentiation, metabolic reprogramming is required to activate relevant cell signaling pathways and supply energy for these biological processes.

### 3.1 Glucose metabolism

Glucose metabolism reprogramming of stem cells controls cell differentiation towards osteoblasts. Glucose uptake is needed for osteoblast differentiation and bone formation. Researchers found that the elimination of glucose transporter 1 (GLUT1) damaged the transport of glucose into cytoplasm and caused decreased osteogenesis [32]. In bone marrow, BMSCs prefer glycolysis instead of oxidative phosphorylation (OXPHOS) as the main energy source to adapt to the anoxic microenvironment and preserve stemness and pluripotency [33]. Nevertheless, as cells differentiate into osteoblasts, metabolic pathways shift from glycolysis to OXPHOS to meet the energy demands of this process, primarily due to the downregulation of hypoxia-inducible factor 1α (HIF-1α) [34]. Additionally, the pentose phosphate pathway (PPP), which is a glycolytic branching pathway, is also activated during the early osteoblast differentiation from BMSCs. Pharmacological suppression of PPP significantly impaired osteogenesis [35]. Mature osteoblasts tend to utilize glycolysis instead of OXPHOS as the main energy source regardless of the oxygen concentration [36-38], though abnormal osteogenesis and osteoporosis also develop due to tricarboxylic acid (TCA) cycle breakdown in osteoblasts [39].

In bone marrow or peripheral blood, RANKL and M-CSF are dominant initiators for osteoclast differentiation from monocytes or macrophages. Nishikawa et al. reported that OXPHOS was enhanced during osteoclast differentiation induced by RANKL [40]. Others argue that RANKL and M-CSF facilitate glycolysis, which is necessary for osteoclast activation and bone resorption [41]. Notably, a coordinated glucose metabolism is essential for precise cell differentiation and a finely tuned skeletal system.

### 3.2 Lipid metabolism

According to a clinical investigation, levels of serum lipids were positively correlated with bone mass loss [42]. Mice with disrupted cholesterol anabolism due to the loss of the *Sc5d* gene, which encodes lathosterol 5-desaturase (an enzyme in the cholesterol synthesis cascade), exhibited downregulated hedgehog (HH) and Wnt/β-catenin signaling pathways. This disruption was accompanied by impaired osteoblast differentiation and reduced bone mass in mandibular bone [43]. Oxidized low-density lipoprotein (OX-LDL) in the serum increases during high-fat feeding, resulting in osteoblast apoptosis through nuclear factor erythroid 2-related factor 2 (NRF2)/nuclear factor kappa B (NF-κB) signaling and osteoporosis in rats [44]. Fatty acid β-oxidation is the metabolic pathway for fatty acid decomposition and energy supply. In fact, fatty acid oxidation is a crucial source of energy in osteoblasts as well. It can provide energy equivalent to 40-80% of the energy produced by glucose metabolism [45]. Additionally, the major energy source for enhanced osteoblast differentiation triggered by PTH is mitochondrial β-oxidation [46]. Carnitine palmitoyl transferase 2 (CPT2) is an enzyme in fatty acid β-oxidation. The beneficial effect of PTH on bone formation and mineralization was eliminated when CPT2 was knocked down in osteoblasts [46]. Additionally, the specific deletion of low-density lipoprotein-related receptor 5 (LRP5) in osteoblasts destroyed bone mass and functional osteoblasts by inhibiting fatty acid oxidation [47].

Cholesterol metabolism and fatty acid β-oxidation are also key regulators of osteoclast differentiation and bone resorption [48]. Researchers have discovered that heat shock protein 90β (HSP90) increased during osteoclast differentiation, bolstering cholesterol biosynthesis and triggering NF-κB signaling, which further led to osteoclast activity and bone resorption [49]. Mitochondrial β-oxidation arises during the osteoclast differentiation stimulated by RANKL and M-CSF [50]. The knockdown of fatty acid transporter 2 (FATP2) by small interfering RNA (siRNA) resulted in inhibited bone resorption and ameliorated osteoporosis, which might be achieved by failed osteoclast differentiation due to β-oxidation impairment and energy insufficiency [51]. By inhibiting osteoclast differentiation, the specific deletion of *Cpt2* in myeloid cells enhanced bone density in female mice [52]. This highlights the importance of β-oxidation in osteoclast differentiation and bone resorption.

### 3.3 Amino acid metabolism

Arrested cell division and impaired osteoblast differentiation occur in MC3T3-E1 cells cultured in the medium without critical amino acids [53]. As for calvarial osteoblasts, the addition of 40 mM glutamate (Glu) to the medium improved the cellular viability and promoted the expression of osteocalcin (Ocn) [54]. Glu is converted to glutamine (Gln) by glutamine synthetase (GS), whereas the opposite reaction is catalyzed by glutaminase (Gls). In human MG-63 osteoblast-like cells under osteogenic induction, the GS activity arises in the first 7 days and then declines. Dexamethasone (Dex) activates the enzymatic activity of GS, while vitamin D and Wnt5A inhibit it, though it is yet unknown how GS activity affects osteogenesis [55]. In contrast, it has been proven that Gls, which is regulated by estrogen-related receptor α (ERRα), promotes the proliferation and osteogenesis of the MSCs and skeletal stem cells (SSCs) and reverses their senescence [56, 57]. Additionally, Wnt/mTOR complex 1 signaling facilitates the osteoblast differentiation of ST2 cells and improves bone mass in mice via activating Gls [58]. Indoleamine-2,3-dioxygenase 1 (IDO1, which catalyzes the conversion of tryptophan to kynurenine) and the kynurenine pathway are required for osteoblast differentiation of human BMSCs [59]. This has been further confirmed by the beneficial role of kynurenic acid on osteogenesis through Wnt/β-catenin signaling [60].

Glutaminolysis is also an essential energy source for osteoclast differentiation and bone resorption. Pharmacological blockade of glutamine transporter alanine-serine-cysteine transporter 2 (ASCT2), or glutaminolysis by V9302 or CB-839 effectively reverses osteoporosis induced by OVX [61, 62]. Furthermore, the activation of the transient serine synthesis pathway and its rate-limiting enzyme phosphoglycerate dehydrogenase (PHGDH) is also indispensable for osteoclast differentiation through epigenetic regulation of the transcription of nuclear factor of activated T cells cytoplasmic 1 (NFATc1), since NFATc1 is an essential transcription factor for osteoclast differentiation and activation [63]. Conversely, L-arginine metabolism catalyzed by arginase-1 exhibits a prominent inhibitory impact on osteoclast differentiation and inflammatory bone erosion [64].

The roles of metabolic pathways in bone metabolism are summarized in Table 1.

## 4. Metabolic changes in osteoporosis

When aging and osteoporosis occur, metabolic reprogramming exists dominantly. BMSCs extracted from aging mice favor OXPHOS as the main energy source, while those from young individuals prefer glycolysis [65]. This might account for decreased proliferation and stemness of senescent BMSCs. Researchers have found that in the bone marrow of mice with osteoporosis, the elevated iroquois homeobox gene 5 (IRX5) suppresses mTOR signaling and results in an OXPHOS barrier, leading to BMSC senescence [66]. This could also disturb the transition from BMSCs toward osteoblasts and hamper osteogenesis, since the metabolic shift towards OXPHOS is essential for the early differentiation of osteogenic progenitor cells [67].

**Table 1:** Metabolic pathways regulate bone metabolism.

Cell Types	Metabolic pathways	Functions	References
**BMSCs**	Glycolysis	Maintain stemness	[33]
**BMSCs**	OXPHOS	Facilitate osteoblast differentiation	[34]
**BMSCs**	Pentose phosphate pathway	Facilitate osteoblast differentiation	[35]
**BMSCs**	Tryptophan metabolism	Facilitate osteoblast differentiation	[59]
**Osteoblasts**	Glycolysis	Facilitate bone formation	[36-38]
**Osteoblasts**	Fatty acid β-oxidation	Facilitate osteoblast differentiation	[45, 46]
**MSCs/SSCs/ST2**	Glutaminolysis	Facilitate cell proliferation and osteoblast differentiation	[56-58]
**Osteoclasts**	OXPHOS	Facilitate osteoclast differentiation	[40]
**Osteoclasts**	Glycolysis	Facilitate osteoclast maturation and bone resorption	[41]
**Osteoclasts**	Cholesterol biosynthesis	Facilitate osteoclast differentiation	[49]
**Osteoclasts**	Fatty acid β-oxidation	Facilitate osteoclast differentiation	[50, 51]
**Osteoclasts**	Glutaminolysis	Facilitate osteoclast differentiation	[61, 62]
**Osteoclasts**	Serine synthesis	Facilitate osteoclast differentiation	[63]
**Osteoclasts**	L-arginine metabolism	Inhibit osteoclast differentiation	[64]

Abbreviations: BMSCs, bone marrow stem cells; OXPHOS, oxidative phosphorylation; MSCs, mesenchymal stem cells; SSCs, skeletal stem cells.

According to a genome-wide analysis, BMSCs extracted from individuals with osteoporosis express more LRP5 mRNA than their healthy counterparts [68]. The increased LRP5 might impair bone mass via fatty acid oxidation as mentioned above. Additionally, abnormal lipid metabolism takes place in senescent osteoblasts induced by serial passage and the serum of osteoporotic patients [69, 70]. Reduced n-3 polyunsaturated fatty acids in senescent osteoblasts might be the cause of the decreased osteogenic ability [69]. The phytoestrogen puerarin and the traditional Chinese medicine Er-Xian Decoction rescue osteoporosis induced by OVX mainly through their benefits on lipid metabolism, such as the improved synthesis of unsaturated fatty acids [70, 71]. This further confirms the indispensable role of coordinated lipid metabolism in skeletal homeostasis.

Apart from glucose and lipid metabolism, the discrepancy of amino acid metabolism also contributes to abnormal bone metabolism and leads to osteoporosis. The differentially expressed amino acids in serum such as leucine, serine, citrulline, cysteine, and tryptophan between osteoporosis patients and their healthy counterparts have been discovered by different teams [72-74]. The traditional Chinese medicine Erzhi Wan has been found to rescue the amino acid metabolic disruption, including serine, alanine, and aspartic acid metabolism, and subsequently boost the bone mass of Sprague-Dawley (SD) rats suffering from osteoporosis [75]. Additionally, after treatment with puerarin, improved valine and leucine metabolism in the serum of OVX rats arises simultaneously with the increased bone mass [71]. When delivered to bone marrow, the induced MSCs undergo osteoblast differentiation and relieve osteoporosis by recovering lipid metabolism and amino acid metabolism of bone [76]. These discrepancies remind us to focus on how amino acid metabolism affects bone homeostasis and the onset of osteoporosis. Kynurenine, which is the end product of tryptophan metabolism, accelerates skeletal aging partly by impeding OXPHOS of osteoblasts [77, 78]. The link between glucose metabolism and amino acid metabolism reminds us of the complexity and precision of the interconnected cell metabolism. In addition to osteoporosis, sarcopenia is also a common disease caused by estrogen deficiency and is usually presented in patients simultaneously with osteoporosis. Similar metabolic disorders, including disturbed glucose, lipid, and amino acid metabolism, occur in the skeletal muscles of OVX mice, emphasizing the role of harmonious metabolism in skeletal health [79].

**Figure 1.**
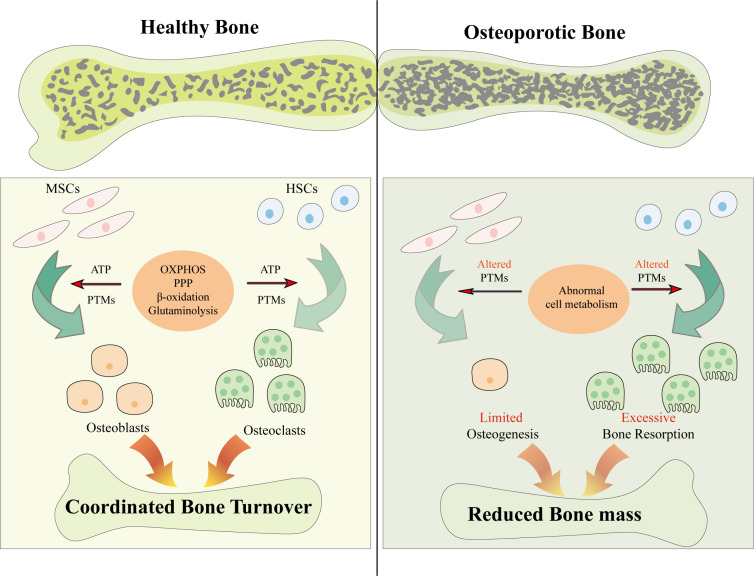
**Osteoporosis results from the metabolic damage**. The bone metabolism is determined by osteoblasts and osteoclasts which are respectively differentiated from MSCs and hemopoietic stem cells (HSCs). During cell differentiation, coordinated cell metabolism pathways supply energy for the proper biological process. Additionally, metabolites originated from cell metabolism can further regulate this process through PTMs. Nonetheless, in osteoporotic bone, the balance between bone formation and resorption has been broken down. Finally, the limited osteogenesis and excessive bone resorption result in reduced bone mass and increased bone fragility. Abbreviations: MSCs, mesenchymal stem cells; HSCs, hemopoietic stem cells; ATP, adenosine triphosphoric acid; PTMs, post-translational modifications; OXPHOS, oxidative phosphorylation; PPP, pentose phosphate pathway.

Except for impaired osteogenesis, excessive osteoclastogenesis is also caused by metabolic reprogramming during osteoporosis. Some scholars point out that OXPHOS is the dominant metabolic pathway in the transition from osteoclast progenitor cells toward osteoclasts, while enhanced glycolysis is indispensable for the following activation of mature osteoclasts and bone resorption [41]. Therefore, folliculin inhibits the expression of cytochrome C and mitochondrial respiration and prevents osteoporosis by hampering osteoclast differentiation [80]. In contrast, nuciferine and the glycolysis inhibitor 2-deoxy-D-glucose (2-DG) alleviate osteoporosis by impeding glycolysis and bone resorption [41, 81]. In bone marrow-derived macrophages (BMDMs) from osteoporotic mice, HIF-1α induces boosted glycolysis and feeble fatty acid oxidation and facilitates bone erosion [82]. However, the specific role of reduced fatty acid oxidation in osteoclasts during osteoporosis has not been fully verified and needs further investigation. Therefore, the metabolic shifts and the metabolite alterations might be the targets to solve or relieve osteoporosis through improving bone turnover (Fig. 1).

## 5. PTMs with metabolites as substrate regulate bone metabolism

Considering the metabolic rewiring that occurs during cell differentiation, we focus on the functions of PTMs mediated by the metabolites in bone metabolism. Thus, we outline the ways in which osteoblast and osteoclast differentiation are regulated by protein acetylation, lactylation, succinylation, palmitoylation, crotonylation, and citrullination.

### 5.1 Protein acetylation in osteoblast and osteoclast differentiation

Since its discovery in 1964, the regulatory role of protein acetylation in many disorders has been extensively investigated [83]. Protein acetylation, encompassing histone acetylation and non-histone acetylation, refers to the process in which acetyl groups transfer from acetyl-CoA to the lysine residues or the N-termini of proteins. Protein acetylation is catalyzed by lysine/histone acetyltransferases (KATs/HATs), also known as “writers” of protein acetylation. KATs mainly consist of the following three protein families: G protein subunit alpha transducin (GNAT), MYST (Moz, Ybf2/Sas3, Sas2, and Tip60), and E1A-binding protein p300/CREB-binding protein (CBP) [84]. In addition, protein acetylation is reversed by lysine deacetylases, including the zinc-dependent histone deacetylases (HDACs) and NAD^+^-dependent sirtuins [84]. Class I (HDAC1, 2, 3, and 8), class II (HDAC4, 5, 6, 7, 9, and 10), and class IV (HDAC11) HDACs are zinc-dependent enzymes, whereas class III HDACs, namely sirtuins, are reliant on the concentration of NAD^+^.

#### 5.1.1 Histone acetylation in osteoblast differentiation

##### 5.1.1.1 Histone acetylation reduces in BMSCs during aging and osteoporosis

During osteoporosis, senescent stem cells exhibit reduced stemness and histone acetylation [85]. The degradation of mitochondrial citrate carriers (CIC) mediated by lysosomes in senescent BMSCs leads to a rapid drop in histone acetylation due to impaired acetyl-CoA transmission from mitochondria to the cytoplasm [86]. In bone marrow, BMSCs reside in a low-oxygen microenvironment. When cultivated at normal oxygen concentrations, BMSCs present reduced histone acetylation, similar to senescent BMSCs, due to the CIC barrier, despite enhanced OXPHOS [87]. Thus, the decreased acetylation at lysine 27 of histone 3 (H3K27ac) in the gene enhancer of *Sp7* causes transcriptional suppression of Osterix, a key transcription factor of osteoblast differentiation, and further impairs osteogenesis [87]. Upregulated nucleosome assembly protein 1 (NAP1) in senescent BMSCs might hamper osteoblast differentiation through epigenetic repression, since NAP1 has been demonstrated to regulate the enrichment of acetylation at lysine 14 of histone 3 (H3K14ac) in the gene promoters of runt-related transcription factor 2 (Runx2), *Sp7*, and *Bglap* (encoding OCN) [88]. Additionally, aging-induced reduction of nicotinamide phosphoribosyl transferase (NAMPT) in osteogenic progenitor cells inhibits acetylation at lysine 9 of histone 3 (H3K9ac), which in turn restricts *Runx2* transcription [89]. Age-related differences in histone acetyltransferases and histone deacetylases are also observed in mice with osteoporosis [86, 90-93]. Generally, PACF, GCN5, CBP/P300, KAT6A, and KAT8 decline in osteoporosis, leading to suppressed histone acetylation and decreased bone mass [91-95].

**Table 2:** Alterations of HATs/HDACs influence osteoblast differentiation.

Enzymes	Alteration in cells	Histone acetylation site	Downstream pathways	Consequence of the alteration	Reference
**P300/CBP associated factor (PCAF)**	Decrease in senescent BMSCs	H3K9ac	BMP signaling	Inhibit osteogenesis	[93]
**GCN5**	Decrease in BMSCs extracted from osteoporotic mice	H3K9ac	Wnt signaling	Inhibit osteogenesis	[92]
**CBP/P300**	Decrease in senescent BMSCs	H3K9ac	β-catenin (Wnt signaling)	Inhibit osteogenesis	[95]
**KAT6A**	Decrease in senescent BMSCs	H3K9ac, H3K14ac	NRF2/NRE signaling	Inhibit osteogenesis	[91]
**KAT8 (Mof)**	Increase in ST2 cells under osteogenic induction	H4K16ac	Runx2 and Ocn	Facilitate osteogenesis	[94]
**C/EBPα**	/	H3K27ac	PPARγ	Facilitate adipogenesis	[96]
**HDAC1**	Increase in osteoblasts from osteoporotic mice	H3ac		Suppress osteogenesis	[100]
**HDAC6, HDAC9**	Increase in senescent BMSCs	H3K9ac, H3K14ac, H4K14ac		Suppress osteogenesis	[98, 99]

Abbreviations: PCAF, P300/CBP associated factor; GCN5, General control nonrepressed 5; CBP, CREB-binding protein; KAT, lysine acetyltransferases; Mof, males absent on the first; C/EBPα, CCAAT/enhancer binding protein α; HDAC, histone deacetylase; BMSCs, bone marrow stem cells; H3K9ac, acetylation at lysine 9 of histone 3; H3K14ac, acetylation at lysine 14 of histone 3; H4K16ac, acetylation at lysine 16 of histone 4, H3K27ac, acetylation at lysine 27 of histone 3; H3ac, acetylation of histone 3; H4K14ac, acetylation at lysine 14 of histone 4; BMP, bone morphogenic protein; NRF2,nuclear factor erythroid 2-related factor 2; NRE, NRF2-replication protein A1 (RPA1) element; Runx2, runt-related transcription factor 2; OCN, osteocalcin; PPARγ, peroxisome proliferator-activated receptor γ.

**Figure 2.**
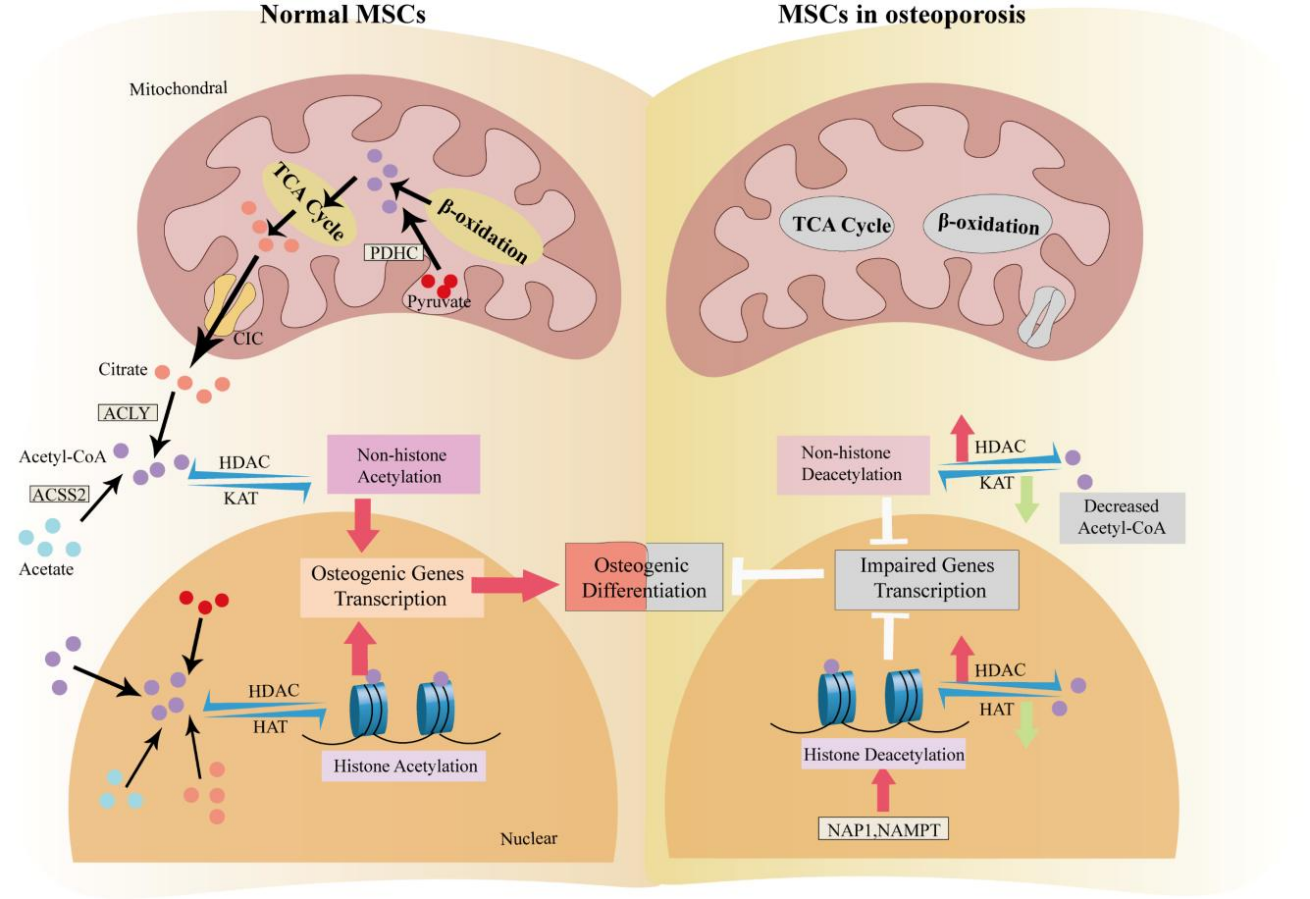
**Impaired osteoblast differentiation in osteoporotic MSCs results from protein deacetylation**. In normal MSCs, acetyl CoA originates from glucose metabolism, β-oxidation and acetate and promotes osteoblast differentiation through protein acetylation. However, the changes of the substrate and enzymes cause protein deacetylation and impaired cell differentiation. Abbreviations: TCA cycle, tricarboxylic acid cycle; PDHC, pyruvate dehydrogenase complex; CIC, citrate carrier; ACLY, ATP-citrate lyase; ACSS2, acyl-CoA synthetase short-chain family member 2; HDAC, histone deacetylase; KAT, lysine acetyltransferases; HAT, histone acetyltransferases; NAP1, nucleosome assembly protein 1; NAMPT, nicotinamide phosphoribosyl transferase.

Specifically, CCAAT/enhancer binding protein α (C/EBPα) recruits H3K27ac to bind the gene promoter of peroxisome proliferator-activated receptor γ (PPARγ), promoting adipogenesis while suppressing osteogenesis [96, 97]. Osteoporosis is also caused by enhanced HDACs, including HDAC1, HDAC6, and HDAC9, in BMSCs [98-100]. The alterations in HATs and HDACs during osteoporosis are summarized in Table 2. MI192, a selective HDAC2/HDAC3 inhibitor, elevates H3K9ac and facilitates osteoblast differentiation in BMSCs [101]. HDAC inhibitors, such as MS-275, suberoylanilide hydroxamic acid (SAHA), trichostatin A (TSA), sodium butyrate (NaBu), and valproic acid (VPA), have also been shown to have a bone-protective function by promoting MSC differentiation into osteoblasts [102-107]. Therefore, restoring histone acetylation in MSCs might be a powerful strategy to restore osteogenesis and rescue osteoporosis (Fig. 2).

##### 5.1.1.2 H3K27ac facilitates osteoblast differentiation

In contrast to trimethylated lysine 27 on histone 3 (H3K27me3), increased H3K27ac is usually considered a marker of transcriptional activation. In SSCs, Pax interaction transcriptional activation domain protein 1 (Ptip) inhibits the H3K27ac enrichment in the gene promoter of glycolytic enzyme phosphoglycerate kinase 1 (Pgk1) and hampers its transcription [108]. Consequently, decreased glycolysis in SSCs helps to maintain the resting state as a reserve cell for osteogenesis rather than immediate proliferation or differentiation. During the early differentiation from BMSCs to osteogenic progenitor cells, the enrichment of H3K27ac instead of H3K27me3 increases in TSS of the *ZBTB16* gene. This facilitates the expression of the encoded promyelocytic leukemia zinc finger (PLZF), which acts as a transcription factor and further enhances H3K27ac through positive feedback [109]. Enhanced long noncoding RNA (lncRNA) OG interacts with HnRNPK in the nucleus to boost the bone morphogenetic protein (BMP) signaling and osteogenesis. Meanwhile, HnRNPK binds to the promoter region of lncRNA OG during the osteoblast differentiation of BMSCs and recruits H3K27ac to activate its transcription [110].

H3K27ac also directly increases the expression of osteogenic proteins like OCN and alkaline phosphatase (ALP) or common osteogenic transcription factors like RUNX2 and Osterix by binding to their gene promoters. In MSCs, c-Jun activated by lncRNA HOTAIRM1 recruits P300 and improves H3K27ac in the *Runx2* gene promoter [111]. Conversely, lncRNA NKILA suppresses NF-κB activation to promote osteoblast differentiation in MSCs, since NF-κB binds to the *Runx2* gene promoter and recruits HDAC2 to inhibit H3K27ac [112]. In MC3T3-E1 cells under osteogenic induction, mitogen-activated protein kinase (MAPK) signaling is activated, and the phosphorylated extracellular signal-regulated kinase (p-ERK) is transported into the nucleus [113]. Then p-ERK induces the recruitment of RNA polymerase II and P300 near the promoter region of *Bglap2* and *Ibsp* (encoding integrin-binding sialoprotein) and promotes gene expression by enhancing H3K9ac and acetylation of lysine 4 on histone 4 (H4K4ac) [113]. Researchers found that the elimination of microRNA-23a cluster (miR-23a) also led to the transformation from H3K27me3 to H3K27ac in the promoters of osteogenic genes and improved bone mass *in vivo* [114]. Additionally, another group has demonstrated that miR-23a inhibited *Ocn* transcription by reducing acetylation at lysine 18 of histone 3 (H3K18ac) and H3K27ac [115]. Surprisingly, the adipogenic transcription factor PPARγ improves H3K27ac in the gene promoter of *Runx2* in periodontal ligament cells (PDLCs) and promotes osteogenesis [116]. In summary, osteoblast differentiation is enhanced when H3K27ac is enriched in osteogenic lineage cells.

##### 5.1.1.3 Mechanical force induces histone acetylation to facilitate osteogenesis

Physically, moderate mechanical stress is beneficial to bone mass and density. Hindlimb unloading (HU) induces osteoporosis by mechanical unloading. In the HU mouse model, increased HDAC1 expression causes H3 deacetylation in the promoter region of the *Jag1* gene [117]. Therefore, reduced expression of *Jag1*, a ligand of the Notch signaling pathway, leads to Notch inactivation and accelerates bone mass loss. The deletion of the oxygen-sensing enzyme proline hydroxylase 2 (PHD2) in osteocytes activates SIRT1, inhibiting *Sost* (encoding sclerostin) gene transcription via reduced H3K9ac enrichment in its promoter region and attenuating osteoporosis in HU mice [118]. The markedly increased general control nonrepressed 5 (GCN5) level in PDLCs induced by mechanical stress triggers *ZBTB16* transcription through elevated H3K9ac and H3K14ac. Thus, enhanced PLZF in turn activates Wnt1/β-catenin signaling, promoting osteogenesis and orthodontic tooth movement (OTM) [119]. On the contrary, in inflammatory PDLSCs, downregulated GCN5 inhibits H3K9ac and H3K14ac in the promoter region of the Dickkopf1 (an inhibitor of Wnt signaling) gene and impairs osteogenesis [120]. Clearly, mechanical loading within physiological parameters is a positive stimulation for histone acetylation and osteogenesis.

##### 5.1.1.4 Histone acetylation mediates osteoporosis induced by prenatal exposure to risks

Prenatal exposure to several dangers is detrimental to the peak bone mass and increases the morbidity of osteoporosis in offspring. Prenatal caffeine exposure leads to elevated serum corticosterone (CORT) levels in offspring rats. CORT binds to the glucocorticoid receptor (GR) and subsequently recruits HDAC11, which inhibits H3K9ac in the promoter region of the 11-beta-hydroxysteroid dehydrogenase (11β-HSD2) gene [121]. Since 11β-HSD2 inhibits excessive glucocorticoids (GC), the epigenetic alteration downregulates the expression of 11β-HSD2 in the bone and increases the susceptibility to osteoporosis in offspring rats. By activating GR, CORT also inhibits IGF1 by reducing H3K9ac and H3K14ac, thereby increasing the morbidity of osteoporosis in subsequent generations [122]. Bisphenol A (BPA) binds to the estrogen receptor β (ERβ) and recruits HDAC5, which further suppresses H3K9ac in the *Tgfb* gene promoter and prevents osteoblast differentiation of osteogenic progenitor cells [123]. Thus, exposure to BPA during pregnancy also raises the incidence of osteoporosis in offspring rats. Prenatal Dex or ethyl alcohol exposure increases H3K9ac and H3K27ac in the promoter region of the angiotensin-converting enzyme (ACE) gene by activating GR or the transcription factor early growth response factor 1 (Egr1) signaling [124, 125]. Increased ACE activates the renin-angiotensin system in the uterus and causes hereditary osteoporosis. Thus, during pregnancy, exposure to risks results in abnormal histone acetylation, causing excessive expression of bone-destructive genes and loss of expression of bone-protective genes. This finally hampers the bone mass, especially the peak bone mass in offspring.

##### 5.1.1.5 Histone acetylation that inhibits osteoblast differentiation

Most evidence mentioned above supports that histone acetylation facilitates osteogenesis. However, histone acetylation can also serve as a negative factor in bone formation under certain conditions. Though necessary for bone homeostasis, excessive GC induces osteoporosis and a high risk of fracture [126, 127]. When GC is added to the culture media of BMSCs, it inhibits osteogenesis and encourages adipogenesis. For example, Dex inhibits osteoblast differentiation in BMSCs under osteogenic induction by reducing RUNX2 expression. This results from the elevated HDAC4 and bromodomain protein 4 (BRD4) as well as reduced H3K9ac in the promoter region of the *Runx2* gene [128]. This result is consistent with the previously described findings. In contrast, under lipid induction, Dex induces the transcription of forkhead box P1 (Foxp1) through elevated BRD4 and H3K9ac in its gene promoter, while incremental Foxp1 acts as the transcription factor of PPARγ and induces adipocyte differentiation instead of osteoblast differentiation [128]. SIRT6 is upregulated during the osteoblast differentiation of DPSCs, leading to reduced H3K9ac and acetylation at lysine 56 of histone 3 (H3K56ac). The application of SIRT6-specific agonist MDL-800 reinforces this histone deacetylation and rescues osteoporosis by improving PPP [129]. While VPA and NaBu have been shown to encourage osteoblast differentiation in MSCs, they also have unintended effects, such as increasing the expression of p21^CIP1/WAF1^ by inducing hyperacetylation of H3 and H4 [130, 131]. This results in cell cycle arrest, proliferative suppression, and abnormal osteoblast differentiation in MSCs. The bidirectional functions of histone acetylation on osteogenesis are worthy of further discussion.

##### 5.1.1.6 Medicine that targets histone acetylation to recover osteogenesis

Researchers have demonstrated that several Chinese medicines, peptides or therapeutic methods promote osteogenesis through histone acetylation. For example, dihydroartemisinin (DHA) was predicted to be a potential treatment for osteoporosis by a deep learning-based efficacy prediction system analysis using RNA-seq data. According to the follow-up study, DHA helps to rescue the stemness, proliferation, and osteoblast differentiation of aging BMSCs, preventing OVX-induced osteoporosis by increasing the expression of GCN5 and H3K9ac [132]. Rosavin is an extract from Rhodiola Rosea. Researchers have shown that rosavin controls bone homeostasis by stimulating osteoblast differentiation and suppressing osteoclast differentiation. This is mediated by reduced HDAC1 and increased H3K9ac in the promoter region of the eukaryotic elongation factor 2 (EEF2) gene [133]. Pomegranate seed oil administered orally reduces HDAC1 expression in rat liver tissues by promoting H3K9ac and H3K14ac in the *Igf1* gene region [134]. Subsequently, the transcription of *Igf1* is activated. New bone formation occurs due to elevated IGF-1 concentrations in serum and bone tissues. By increasing H3K9ac in the promoter region of genes in Wnt signaling (including *Wnt3a* and Disheveled 3 (DVL3)), ferutinin, a polycyclic phytoestrogen extracted from *Ferula*, facilitates the activation of the Wnt pathway and osteoblast differentiation of DPSCs [135]. MOZ and MORF are essential HATs in PDLSCs. Osthole, a native compound derived from traditional Chinese herbs, has been demonstrated to restore decreased H3K9ac, H3K14ac, and osteogenesis in the inflammatory PDLSCs by upregulating the expression of MOZ and MORF [136]. Additionally, electroacupuncture has been found to restore bone mineral density and structure by downregulating HDAC2 and upregulating Ac-H3 [28]. Clearly, it seems quite feasible to rescue bone mass by improving histone acetylation.

#### 5.1.2 Non-histone acetylation in osteoblast differentiation

Non-histone acetylation can facilitate osteogenesis by regulating the stability and activity of several transcription factors in stem cells. As an essential transcription factor for osteoblast differentiation, RUNX2 acetylation plays an important role in maintaining its stability and function. In C2C12 cells stimulated by BMP2, activated Smad1/5 facilitates the expression of P300 binding to RUNX2 and results in RUNX2 acetylation. Since the specific lysine residue of RUNX2 is occupied by acetyl groups, ubiquitin binding to RUNX2 decreases. This prevents RUNX2 from degradation through the ubiquitin-proteasome system mediated by Smad ubiquitin regulatory factor 1 (Smurf1, an E3 ubiquitin ligase), and encourages osteoblast differentiation [137]. Thus, by preserving RUNX2 acetylation and its transcriptional activity, HDAC inhibitors, including TSA, SCOP402, MS-275, and SAHA, can be utilized to preserve bone mass [102, 138]. Similar expression to FGF genes (Sef) is a ligand for fibroblast growth factor receptor and serves as a competitive inhibitor of fibroblast growth factor 2 (FGF2). During the osteogenic induction of osteoblasts, bone marrow cells, and MSCs, elevated Sef expression conversely leads to considerable RUNX2 deacetylation and osteogenic damage, which can be prevented by TSA [139]. In MC3T3-E1 cells, p300/CBP-associated factor (PCAF) has been shown to directly bind to RUNX2 and catalyze its acetylation [140]. In the bone tissues of HU rats, increased miR-132-3p targets the 3’-UTR of the *EP300* mRNA and inhibits its translation, which further results in RUNX2 deacetylation and ubiquitination and damages bone formation [140, 141]. Conversely, in BMSCs, miR-29a contributes to maintaining RUNX2 acetylation that can be reversed by the elevated HDAC4 under the stimulation of GC [142]. The overexpression of miR-29a clearly has a positive effect on bone mass. The aforementioned findings provide credence to the idea that RUNX2 acetylation and osteoblast differentiation are favorably connected, while other researchers hold the opposite standpoint. Uncarboxylated osteocalcin (unOC) is a regulator of energy and carbohydrate metabolism. It has been shown that unOC activates SIRT1 through protein kinase A (PKA)/adenosine monophosphate-activated protein kinase (AMPK) signaling in BMSCs [142]. This further suppresses RUNX2 acetylation while strengthening osteoblast differentiation rather than adipogenesis [142]. The addition of resveratrol to the medium of ADMSCs also activates the SIRT1/RUNX2 deacetylation axis and exhibits an effect on improving osteoblast differentiation [143]. Apart from RUNX2, Osterix also undergoes acetylation at lysine 307 and 312. The acetylation of Osterix is catalyzed by CBP/p300 and eliminated by HDAC4. This process promotes the stability of Osterix in osteogenic progenitor cells and enhances the transcriptional activity of downstream genes to enhance osteoblast differentiation [144]. Wnt/β-catenin signaling is indispensable for osteoblast differentiation. During the osteoblast differentiation of C3H10T1/2 cells, the acetylation of β-catenin increases as a result of improved OXPHOS activity and acetyl CoA synthesis [145]. There appears to be a positive correlation between osteoblast differentiation and β-catenin acetylation. Nevertheless, it has also been demonstrated that the deacetylation of β-catenin catalyzed by SIRT1 facilitates osteoblast differentiation of MSCs and DPSCs [146, 147]. When SIRT1 was specifically deleted in aged mice, the β-catenin acetylation at lysine 49 and 345 increased. Nonetheless, decreased nuclear translocation of β-catenin occurs due to the enhanced acetylation, leading to a more severe osteoporotic phenotype [146]. Therefore, more research is necessary to explain the disparity.

The Forkhead transcription factor O (FOXO) protein family participates in many biological processes such as cell proliferation, apoptosis, oxidative stress response, and metabolism, and mediates bone turnover and osteoporosis [148]. In C2C12 cells, exogenous stimuli activate AMPK signaling, altering the ratio of NAD^+^/NADH and SIRT1 activity [149]. Therefore, active SIRT1 catalyzes the deacetylation of PPARγ coactivator 1-α (PGC1α), FOXO1, and FOXO3. This may explain why AMPK and SIRT1 coordinately regulate various biological processes [149]. Resveratrol prevents cell death and enhances the antioxidant capacity of osteoblasts via activating SIRT1 and deacetylation of FOXO1, thereby mitigating OVX-induced osteoporosis [150]. This is further confirmed by the bone-protective role of resveratrol in the isolation-based mouse anorexia model (SBA). By activating SIRT1, resveratrol prevents FOXO1 and RUNX2 acetylation, restores osteoblast differentiation in BMSCs isolated from SBA mice, and rescues bone density *in vivo* [151]. The acetyltransferase responsible for FOXO1 acetylation is CBP/p300. Thus, FOXO1 deacetylation can be facilitated by the specific inhibitors of CBP/p300. The deacetylated FOXO1 subsequently translocates into the nucleus, where FOXO1 serves as a transcription factor and stimulates the transcription of signal transducer and activator of transcription 1 (STAT1) [95]. Elevated STAT1 inhibits osteogenesis through impacts on the chemokine signaling pathway. The deletion of B-cell-specific Moloney mouse leukemia virus integration site 1 (Bmi-1) leads to an osteoporotic phenotype in mice as a result of reduced SIRT1 expression and elevated FOXO3 acetylation. This impairs the expression of downstream antioxidants, including superoxide dismutase 2 (SOD2) [152]. The deacetylation of SOD2 enhances its antioxidant properties. For example, irisin increases SIRT3 expression through the activation of the AMPK-PGC-1α signaling pathway. Activated SIRT3 induces deacetylation of SOD2 at lysine 68 (K68), which restores mitochondrial antioxidant capacity in ASCs, reverses stem cell injury caused by advanced glycosylation end products and stimulates their osteoblast differentiation [153]. Additionally, in MC3T3-E1 cells, endogenous melatonin improves the expression of SIRT3, which reduces the Ac-SOD2/SOD2 ratio, alleviates mitochondrial oxidative damage, and promotes osteoblast differentiation [154]. This further promotes the formation of new bones around the prosthesis in osteoporotic rats. Patients with osteoporosis have elevated serum levels of FOXO4. FOXO4 acetylation catalyzed by CBP in BMSCs suppresses Wnt/β-catenin signaling, impairs osteoblast development, and exacerbates osteoporosis [155].

In addition to its involvement in osteoclast differentiation, NF-κB signaling also regulates osteoblast differentiation. The specific function is influenced by the acetylation of p65, which is a key component of NF-κB and an essential transcription factor. Downregulated p65 acetylation at lysine 310 (K310ac) caused by SIRT6 in BMSCs has been shown to promote osteoblast differentiation and ectopic bone formation [156]. Interferon-related developmental regulator 1 (IFRD1) is an early response gene that responds rapidly to stimuli from numerous signals. Additionally, IFRD1 inhibits osteogenesis through its dual function on p65 and β-catenin acetylation [157]. On the one hand, IFRD1 promotes the interaction between HDAC1 and p65, leading to p65 K122 and K123 deacetylation and nuclear translocation. Consequently, the transcription of the downstream *Smad7* increased, seriously impairing phosphorylation of Smad1/5/8 and the expression of Osterix to harm osteogenesis. On the other hand, IFRD1 promotes the connection between HDAC1 and β-catenin, which results in the deacetylation at β-catenin K49 and its translocation to the cytoplasm. This in turn suppresses the transcription of osteoprotegerin (OPG) and the differentiation of osteoblasts. Researchers have also found that in osteoblasts, the stimulation of bFGF enhances the interaction between p300 and NF-κB, promoting P50 acetylation and the transcription of target genes [158]. However, more studies are required to determine whether P50 acetylation affects osteogenic pathways. p53 is an acknowledged indicator of cellular senescence, and GTDF is a flavanol isolated from *Ulmus wallichiana*. By inhibiting p53 acetylation and expression in osteoblasts, GTDF shields osteoblasts from apoptosis induced by Dex and methylprednisolone and alleviates osteoporosis caused by GC [159]. Acetylation of p53 at lysine 382 (K382) increases in rats with osteoporosis induced by cadmium. Thus, overexpressing SIRT1 may serve as a therapeutic strategy to inhibit p53 acetylation and restore bone mass [160]. PPARγ is a significant transcription factor for adipogenesis. By suppressing SIRT1 activity, GC and the neuropeptide regulated by GC cause PPARγ acetylation and reduce its ubiquitination degradation [161]. Thus, an overabundance of PPARγ results in a phenotype that promotes adipogenesis while inhibiting osteogenesis. In osteoblasts, SIRT1 also induces deacetylation at lysine 381 of glutamine oxaloacetic aminotransferase (GOT1) and its inactivation. This subsequently enhances glycolysis and increases osteoblast differentiation [162]. After deleting SIRT1 in osteoblasts, decreased bone density was observed in mice. However, the addition of aminooxyacetic acid (AOA), an acknowledged inhibitor of GOT1, recovered bone mass [162]. In the OTM model, stress could enhance the expression of KAT6A in PDLSCs. Subsequently, KAT6A binds to Yes-associated protein (YAP) and catalyzes its acetylation [163]. YAP deacetylation regulates alveolar bone resorption by regulating the ratio of RANKL/OPG through the YAP/transcription enhancer-associated domain 4 (TEAD4) axis. Nonetheless, the impact of YAP acetylation on osteoblast or osteoclast differentiation has not been fully confirmed. The 14-3-3 protein family regulates physiological activities and collaborates with multiple pathways. Acetylation at K49 and K51 of the 14-3-3 protein inhibits its phosphorylation and leads to its inactivation. Elevated levels of 14-3-3β K51ac, catalyzed by HBO1 (also known as KAT7), are detected in the cytoplasm and nucleus of ASCs during osteoblast differentiation [164]. However, the specific regulatory role of 14-3-3 acetylation in stem cell differentiation has not been clearly clarified.

#### 5.1.3 Histone acetylation in osteoclast differentiation

Histone acetylation can also regulate osteoclast differentiation. The addition of 1,25(OH)_2_D_3_ induces a significant increase in C/EBPβ and histone 4 acetylation (Ac-H4) in the promoter region of the *Rankl* gene in bone stromal cells. As a result, RNA polymerase Ⅱ could readily bind to the TSS of the *Rankl* gene, facilitating the secretion of RANKL and osteoclast differentiation [165, 166]. Additionally, the bromo and extra-terminal protein (BET)-specific inhibitor I-BET115 rescues osteoporosis and arthritis by epigenetically inhibiting osteoclast differentiation. Specifically, I-BET115 inhibits the expression of the transcription factor MYC. Thus, declining MYC levels results in the decreased recruitment of CBP as well as Ac-H4 in the promoter region of the *Nfatc1* gene [167]. This further damages its transcriptional activity and subsequent osteoclast differentiation. Nuclear receptor corepressor (NCoR) was recognized as a transcriptional corepressor, which functioned as the NCoR/HDAC3 complex that hampered transcription by inducing H3K27 deacetylation [168]. However, recently it has been shown that PPARγ coactivator 1-β (PGC1β) interacts with the dimer and forms the NCoR/HDAC3/PGC1β complex in osteoclasts under the stimulation of RANKL. The complex reverses the transcription-repressive role of the dimer and induces elevated H3K27ac enrichment in the promoter region of osteoclastic genes, such as acid phosphatase 5 (ACP5) and OC-STAMP, further promoting osteoclast differentiation [169]. Actually, after the formation of the complex, enhanced HDAC3 activity is consumed through PGC1β deacetylation [170]. Meanwhile, RANKL also recruits p300 in these gene promoters and facilitates H3K27ac in the TSS of the downstream genes [170]. The H3K18ac catalyzed by CBP/p300 can also serve as an MMP-9 recognition and cleavage site, leading to the hydrolysis of the N-terminal tail of histone 3 during osteoclast differentiation. This enhances DNA accessibility and promotes the expression of NFATc1, leukemia inhibitory factor (LIF), and xenotropic and polytropic retrovirus receptor 1 (XPR1) [171].

Histone acetylation also links to several pathways that are essential for osteoclast differentiation. In osteoporotic mice induced by OVX, miR-181 (an antagonist for KAT2B) is reduced, leading to enhanced KAT2B activity in both BMSCs and BMDMs. This stimulates serine- and arginine-rich splicing factor 1 (SRSF1) gene transcription by increasing H3K27ac enrichment in its promoter region [172]. The KAT2B/H3K27ac/SRSF1 axis shows the dual effect of inhibiting osteoblast differentiation and promoting osteoclast differentiation, thus aggravating osteoporosis. The expression of SET domain-containing protein 2 (SETD2) increases during the osteoclast differentiation of macrophages. Elevated SETD2 promotes the enrichment of H3K9ac and acetylation at lysine 8 of histone 4 (H4K8ac) in the promoter region of the *Wnt5a* gene. Subsequently, osteoclast activation is promoted as a result of the activated noncanonical Wnt pathway [173]. As a key enzyme in adipose de novo synthesis, ATP-citrate lyase (ACLY) links cellular metabolism to histone acetylation [174]. During osteoclast differentiation induced by RANKL, the enzymatic activity of ACLY increases and the downstream metabolite acetyl-CoA accumulates in the nucleus. Increased acetyl-CoA enhances the acetylation levels at several histone sites, including H3K9ac, H3K27ac, H3K18ac, H3K14ac, and acetylation at lysine 23 of histone 3 (H3K23ac) [174]. Therefore, the loose chromatin leads to enhanced gene transcription of *Rac1* and osteoclast differentiation. In contrast, ACLY-specific inhibitor BMS-303141 hampers osteoclast differentiation and rescues the bone mass in OVX mice by histone deacetylation and chromatin compaction [175, 176]. RAC1 regulates cytoskeletal formation and bone resorption activity of osteoclasts through phosphatidylinositol 3-kinase (PI3K)/protein kinase B (AKT) signaling [176]. During osteoclast differentiation, the negative relationship between Krüppel-like factor 2 (KLF2) and autophagy has been verified. RANKL reduces KLF2 while increasing H3K9ac and H4K8ac in the TSS of the *Becn1* gene, which encodes an autophagy protein, thereby enhancing autophagy [177]. Additionally, overexpressed KLF2 reduces arthritis, suggesting the important role of histone acetylation and autophagy controlled by KLF2 in bone resorption. Overall, there is an evident positive link between histone acetylation and osteoclastogenesis.

#### 5.1.4 Non-histone acetylation in osteoclast differentiation

It has been reported that RANKL promotes osteoclast differentiation from bone-derived cells by inducing p300 expression and NF-κB p65 acetylation. Since SIRT1 catalyzes p65 deacetylation, the decline in SIRT1 in the bone tissue of aged mice causes increased acetylation of p65 K310, which triggers bone resorption and inhibits bone formation [178]. As a natural agonist of SIRT1, resveratrol facilitates the formation of SIRT1/p300/p65 complex, causing p65 deacetylation and nuclear export to inhibit osteoclastic gene transcription [179]. The endogenous steroid cholesterol sulfate (CS) also activates SIRT1 via AMPK signaling [180]. Therefore, CS inhibits *Nfatc1* transcription due to K310 deacetylation, preserving bone density from osteoporosis caused by OVX or inflammatory bone resorption triggered by lipopolysaccharide (LPS). In BMDMs extracted from OVX mice, increased miR-128 targets 3’-UTR of the *Sirt1* mRNA and induces its degradation [181]. Decreased SIRT1 results in bone resorption by increasing p65 K310ac. Carpio et al. found elevated NF-κB acetylation in HDAC3-deficient chondrocytes, triggering the production of matrix-degrading proteins such as IL-6 and MMP-13 and promoting osteoclast differentiation via paracrine signaling [182]. In BMDMs, RANKL induces PCAF activity and promotes the acetylation of the transcription factor NFATc1 to reinforce its protein stability and the downstream transcription of tartrate-resistant acid phosphatase (TRAP) [183]. Conversely, HDAC5 and HDAC6 catalyze the deacetylation of NFATc1. Additionally, RANKL promotes the connection between p300 and PCAF as well as the acetylation of PCAF regulated by p300. The acetylation of PCAF further increases its enzymatic activity [183]. The synthetic SIRT1 agonists SRT2104 and SRT3025 stimulate FOXO1 deacetylation, enhancing the transcription of downstream heme oxygenase-1 to inhibit osteoclast differentiation via mitochondrial respiratory chain disruption [184]. Researchers have also found that SRT2183 and SRT3025 block osteoclast differentiation through SOD2 K68 acetylation, which is likely caused by decreased SIRT3 expression, although the precise mechanism is not yet known [185]. Mitophagy-related proteins, including PTEN-induced kinase 1 (PINK1) and ATPase inhibitory factor 1 (ATPIF1), have been found to be acetylated in myeloid cells after specific deletion of SIRT3 [186]. This suggests that SIRT3 may affect mitophagy by protein acetylation to inhibit osteoclast differentiation and bone resorption. The adaptor protein p66Shc mediates cell apoptosis in a ROS-dependent manner. High glucose and palmitic acid facilitate p66Shc acetylation by inhibiting SIRT1 expression [187]. Thus, enhanced acetylation of p66Shc promotes its phosphorylation on Ser36 and ROS synthesis, indirectly activating NF-κB signaling to promote osteoclast differentiation [187].

**Figure 3.**
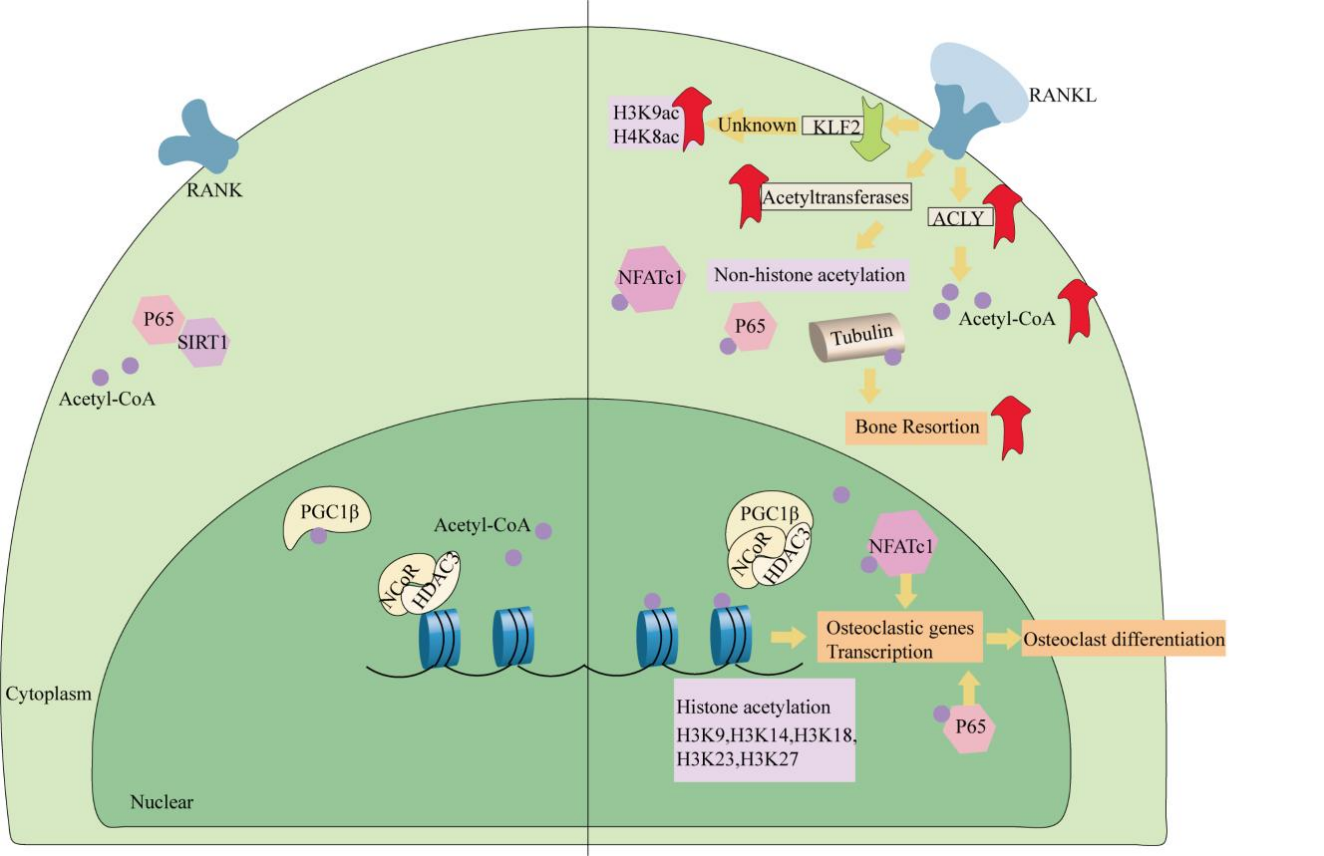
**RANKL induces protein acetylation to facilitate osteoclast differentiation**. RANKL induces the formation of NCoR/HDAC3/PGC1β complex in osteoclasts. Therefore, histone acetylation arises since the deacetylase activity of HDAC3 has been consumed by PGC1β. Additionally, RANKL also induces the production of acetyl CoA and activates KATs. Therefore, osteoclast differentiation is improved by the elevated protein acetylation. Abbreviations: RANKL, receptor activator of nuclear factor kappa-B ligand; RANK, receptor for RANKL; KLF2, kruppel-like factor 2 [lung]; ACLY, ATP-citrate lyase; NFATc1, nuclear factor of activated T cells, cytoplasmic 1; PGC1β, PPARγ coactivator 1-β; NCoR, nuclear receptor co-repressor; HDAC3, histone deacetylase 3; SIRT1, sirtuin 1.

The function of bone resorption in osteoclasts depends on microtubule networks that are controlled by tubulin acetylation. In RAW264.7 cells, RANKL induces glycogen synthase kinase 3β (GSK3β) Ser9 phosphorylation. The phosphorylated GSK3β interacts with hnRNPK (a member of the DNA/RNA-binding to heterogeneous nuclear ribonucleoprotein (hnRNP) family) in the cytoplasm to regulate α-tubulin acetylation, which determines NFATc1 expression and osteoclast maturation [188]. Researchers have also discovered that AKT activates GSK3β and upregulates tubulin acetylation to sustain the bone resorption ability of osteoclasts [189]. In addition, by improving tubulin acetylation, calpain-6 reverses the cytoskeleton instability in osteoclasts caused by Dex [190]. The Rho-mDia2 axis is responsible for the arrangement of F-actin and regulates podosome assembly. Researchers have demonstrated that the Rho-mDia2 axis activates HDAC6 to promote the deacetylation of tubulin. Deacetylation of tubulin in osteoclasts impairs its stability, hinders the formation of the podocyte band, and then inhibits the adhesion of osteoclasts to surrounding tissues and matrix degradation [191]. In OVX mice, Parkin binds to HDAC6 to facilitate α-tubulin deacetylation and restores bone mass [192]. Furthermore, during osteoclast differentiation from BMDMs, the deficit of tetraspanin 7 prevents α-tubulin acetylation [193]. The following breakdown of the cytoskeleton impedes the formation of closed areas and bone resorption. Obviously, tubulin acetylation is indispensable for osteoclast differentiation, maturation, and function (Fig. 3).

#### 5.1.5 Biological materials that target acetylation to facilitate osteogenesis

Given the critical role that acetylation plays in osteogenesis and bone resorption, the biological materials targeting acetylation might be potent strategies to restore bone density in several bone diseases, including osteoporosis. Protein acetylation is mainly regulated by either the substrate acetyl-CoA or the associated enzymes. By activating Piezo1 ion channel, an ion channel and a mechanical sensor on the cell membrane, TiO_2_ nanotubes improve OXPHOS in BMSCs [194]. Thus, the enhanced OXPHOS flux promotes the buildup of acetyl-CoA, the substrate for β-catenin acetylation, thereby enhancing BMSC osteogenesis by increasing acetylated β-catenin. The nucleus senses mechanical stress and control cellular fate. When cultivated in photo-stiffening hydrogels, nuclear tension increases in MSCs due to enhanced hydrogel stiffness caused by a photo-initiated reaction [195]. Thus, HDACs apparently decrease, leading to epigenetic remodeling, which is due to the elevated histone acetylation [195]. The elevated histone acetylation contributes to the expression of RUNX2 and facilitates osteoblast differentiation. Additionally, introducing metabolites of the TCA cycle to biomimetic hydrogels improves the mechanical and anti-swelling properties of hydrogels. Except for altering the material properties, the addition of citrate also enhances the TCA cycle and the generation of acetyl-CoA, which serves as the substrate for histone acetylation and elevates the H3K9ac [196]. Thus, due in part to the epigenetic alteration, the biohydrogels containing citrate greatly promote bone repair.

EVs are thought to be potent carriers for regenerative medicine [197]. Researchers have found that EVs could transfer epigenetic information between donor cells and recipient cells. In MC3T3-E1 cells, exogenous TSA causes a substantial reduction in HDACs, and this epigenetic alteration can be passed on to the EVs (TSA-EVs) secreted by MC3T3-E1 cells [198]. Co-culturing BMSCs with TSA-EVs results in higher H3K9ac and greater extracellular matrix mineralization compared to BMSCs co-cultured with EVs from osteoblasts untreated with TSA [198]. This confirms the importance of EVs in epigenetic information transmission. According to another study, EVs isolated from BMSCs cultivated with 5-azacytidine (5-AZA) or deferoxamine (DFO) similarly impart increased histone acetylation to the recipient cells, such as BMSCs and HUVECs, and modify their biological functions [199]. Gelatin methacryloyl (GelMA) hydrogels functionalized with synthetic Laponite nanoclay (LAP) could encapsulate TSA-EVs and enhance their beneficial effect on osteoblast differentiation of BMSCs by manipulating EV release [200].

The impact of physical and chemical stimuli on histone acetylation has been mentioned above. In order to determine the cell fate of ADSCs, the programmable shape-memory polymer actuator (SMPA) sheets alter the mechanical and thermal microenvironment and regulate H3K9ac through Ca^2+^ influx and downstream signals in ADSCs [201]. The 3D collagen scaffolds incorporate the MSCs that had undergone the epigenetic alteration induced by NaBu and serve as scaffolds for tissue regeneration [202]. Obviously, linking PTMs to biological materials is a powerful bone tissue engineering technique that requires more investigation.

### 5.2 Protein lactylation in osteoblast and osteoclast differentiation

Protein lactylation is defined as the addition of lactate to the lysine residue of histones and non-histones. Since its first discovery in 2019 by Zhang et al., protein lactylation has been regarded as an important type of PTM that regulates gene expression, protein function, and diseases [203]. It has been discovered that lactylation, with lactyl-CoA as the substrate, controls the osteoblast differentiation of stem cells. During the development of teeth and alveolar bone in embryos, lysine lactylation (pan-Kla) is extensively expressed in epithelial-derived cells, interstitial-derived cells, and adjacent bone tissue, indicating that protein lactylation may control the development of these mineralized tissues [204]. Active glycolysis triggered by high-glucose environments during BMP2-induced osteoblast differentiation in C2C12 cells raises lactate, which in turn increases lactylation and osteogenesis [205]. Oxamate is an inhibitor of lactate dehydrogenase A (LDHA), and p300 is the writer of lactylation. Thus, the addition of oxamate and the specific deletion of p300 reduce lactylation and harm osteogenic gene expression [205]. In C2C12 cells, the reduced osteoblast differentiation under low-glucose culture conditions is restored by lactate supplementation. This further confirms the crucial role of lactate-based lactylation in the process. Furthermore, researchers have discovered that in osteogenic progenitor cells, LDHA knockdown via short hairpin RNA (shRNA) reduces the lactylation at histone 3 lysine 18 (H3K18la) in the promoter region of the *JunB* gene, linking histone lactylation to osteoblast differentiation through JunB signaling [206]. In BMSCs extracted from rat alveolar bone, mechanical stretches induce histone lactylation to regulate the proliferation, migration, and osteoblast differentiation of BMSCs, affecting bone remodeling during orthodontic tooth movement [207]. According to our research group, lipopolysaccharide (LPS) inhibits osteoblast differentiation of PDLSCs by preventing protein lactylation. Proanthocyanidin, a natural polyphenol compound, restores the osteogenic capacity of PDLSCs by recovering the decreased lactylation level [208]. Additionally, we hypothesize that Wnt/β-catenin signaling might be the downstream pathway regulated by lactylation in PDLSCs. Further research is required to validate the idea.

Protein lactylation also depends on the lactate in the extracellular matrix in addition to endogenous lactate, since lactate could be transferred among various cells by monocarboxylate transporters on the cell membrane. In vascular endothelial cells (ECs) extracted from bone marrow of patients with osteoporosis, the expression of pyruvate kinase 2 (PKM2) decreases, leading to decreased lactate synthesis and secretion [209]. When co-culturing ECs with BMSCs, the reduced lactate suppresses H3K18la in BMSCs and inhibits the H3K18la enrichment in the promoter region of osteogenic genes, including *Col1a2*, *Comp*, *Enpp1*, and *Tcf7l2* [209]. Therefore, reduced osteoblast differentiation and aggravated osteoporosis result from osteogenic damage caused by the reduction of H3K18la. In bone tissue of patients with osteoporosis, reduced crystallin protein αB (CRYAB) causes increased degradation of the iron homeostasis key protein 1 (FTH1) through the proteasome pathway. Additionally, sodium lactate stabilizes FTH1 similarly to CRYAB, whereas the glycolysis inhibitors 2-DG and oxamate promote its degradation [210]. Since FTH1 contributes to the cell viability and osteoblast differentiation of BMSCs, further research is necessary to determine the possible role of lactylation on FTH1 stability. In addition to the pan-Kla or histone lactylation, researchers have also confirmed the existence of non-histone lactylation, which facilitates protein stability in a way similar to acetylation. As an anticholinergic agent, scopolamine induced the lactylation of RUNX2 at lysine 176 and enhanced the protein stability through deubiquitination, which significantly improved osteoblast differentiation of PDLSCs [211]. Taking the similarities between acetylation and lactylation into consideration, it is worthwhile exploring the lactylation of other transcription factors that participate in bone homeostasis maintenance and are regulated by acetylation.

### 5.3 Protein succinylation in osteoblast and osteoclast differentiation

Zhao and colleagues discovered protein succinylation in 2010 and hypothesized that it might play a role in regulating cell functions [212]. A succinylation proteomic analysis was conducted to evaluate variations in succinylation levels among postmenopausal women across the three groups: osteoporosis, osteopenia, and normal groups. The analysis revealed distinct patterns of succinylated protein expression in each group [213]. This suggested that the decreased bone mass might be caused by the differentially expressed succinylated proteins. Furthermore, thyroid hormones integrate with their succinylated receptors and activate ERK and Akt, thereby promoting osteoblast proliferation [214]. Enzymes in the TCA cycle and fatty acid metabolism could also be succinylated [215]. However, it remains unknown whether such modifications influence metabolic activities and bone turnover.

### 5.4 Protein palmitoylation in osteoblast and osteoclast differentiation

According to certain studies, saturated fatty acids, such as palmitic acid, might induce osteoblast death via autophagy and apoptosis and impair osteogenesis [216, 217]. Palmitic acid (PA) is one of the fatty acids most abundantly released by adipocytes in bone marrow and could cause oxidative stress, endoplasmic reticulum stress, and apoptosis, all of which impair osteoblast functions. Palmitoyl S-acyltransferases (PATs) catalyze a reversible PTM, namely S-palmitoylation. It refers to the addition of long-chain palmitic acid to cysteine residues through labile thioester linkages [218]. The essential PATs in mammals are the zinc finger DHHC domain-containing family (ZDHHCs). ZDHHCs consist of at least four transmembrane domains and the Asp-His-His-Cys (DHHC) pattern. Additionally, protein depalmitoylation is catalyzed by acyl-protein thioesterase 1 (APT1).

At least 24 ZDHHCs have been discovered in mouse calvarial osteoblasts, which raises the possibility that protein palmitoylation plays a part in osteogenesis [219]. Protein palmitoylation promotes the expression of Osterix to strengthen osteoblast differentiation, while the palmitoylation inhibitor 2-bromopalmitate (2-BP) significantly reverses the process [219, 220]. As a palmitate acyltransferase, porcupine serves as a ligand of the Wnt signaling pathway and contributes to the bone mass in both compact and spongy bone [221]. 1,25(OH)_2_D_3_ enhances the expression of ZDHHC1, ZDHHC2, and ZDHHC12 [222]. In order to determine the precise role of ZDHHC13 in the organism, researchers bred *Zdhhc13*-mutant mice, which exhibited the phenotype of osteoporosis, alopecia, shortened lifespan, and multiple organ amyloidosis [223]. Specifically, osteoporosis results from delayed development of the secondary growth center in the epiphyseal plates of long bones and aberrant intrachondral ossification due to decreased palmitoylation of the membrane-type 1-matrix metalloproteinase (MT1-MMP) [224]. MT1-MMP palmitoylation catalyzed by ZDHHC13 regulates the expression of vascular endothelial growth factor (VEGF) in ATDC5 cells and the expression of OCN in MC3T3-E1 cells [224]. In fact, the level of VEGF in epiphysis regulates its vascularization, which is essential for intrachondral ossification and the formation of secondary growth centers.

The palmitoylation of several receptors on cell membranes controls their localization and functions. Osteoporosis in postmenopausal women is mostly caused by a lack of estrogen, as estrogen protects bone by binding to the specific receptors, such as ERα and ERβ [225]. The palmitoylation of ERα C451 (the 451^st^ cysteine) in mice (which corresponds to ERα C447 in humans) affects both its localization on the membrane and its activity. The bone-protective function of estrogen is lost in OVX mice with C451 loss-of-function mutants [226]. IFN-induced transmembrane protein (IFITM) is a family of IFITM-like transmembrane receptors confined to bone. Researchers have discovered three palmitoylation sites in IFITM5, including C52, C53, and C86 [227]. The IFITM5 C52/53 mutation causes improper subcellular localization outside the cell membrane due to depalmitoylation. The depalmitoylation of C52/53 might be a likely cause of type V osteogenesis imperfecta induced by the IFITM5 mutant, though the exact mechanism has not yet been confirmed. APT1 catalyzes the depalmitoylation of bone morphogenetic protein receptor 1α (BMPR1α). Thus, it promotes osteoblast differentiation by triggering the BMP/Smad signaling cascade, alleviating senile osteoporosis [228].

Additionally, it has been established that a number of proteins undergo palmitoylation during osteoclast differentiation induced by RANKL. Despite its inhibitory effect on MC3T3-E1 osteoblast differentiation, 2-BP also pharmacologically inhibited osteoclast differentiation by inhibiting the expression of the key transcription factors c-Fos and NFATc1 *in vitro* [229]. As a result, 2-BP synthetically restored bone mass in OVX mice. Further research is necessary to determine the specific mechanism of palmitoylation regulating bone metabolism.

### 5.5 Protein crotonylation in osteoblast differentiation

Protein crotonylation was discovered in 2011 by Tan and his colleagues [230]. Crotonyl CoA, the substrate of protein crotonylation, is converted from crotonate by acyl-CoA synthetase short-chain family member 2 (ACSS2). A favorable correlation between protein crotonylation and the osteoblast differentiation of PDLSCs has been confirmed. Researchers have found that protein crotonylation might positively correlate with osteogenesis, as its level increases during osteoblast differentiation of PDLSCs [231]. Specifically, as predicted by the crotonylation proteomic analysis, protein crotonylation might regulate osteoblast differentiation by PI3K/AKT signaling. In contrast to the completely opposing impact of deleting ACSS2, exogenous NaCr dramatically increases crotonylation in PDLSCs, stimulates PI3K/AKT signaling, and promotes osteoblast differentiation. Unfortunately, the role of protein crotonylation in bone mass regulation has not been fully investigated in this study. Thus, we anticipate that future research will concentrate on how protein crotonylation affects bone turnover and diseases.

### 5.6 Protein citrullination in osteoblast and osteoclast differentiation

According to a clinical survey, the level of citrulline in the serum of osteoporosis patients was significantly higher than that in healthy control groups, indicating that the elevated citrulline might be a potential diagnostic biomarker of osteoporosis [74]. Elevated citrulline has also been discovered in patients with chronic kidney disease and Alzheimer disease, which prompts us to investigate the impact of citrulline on age-related diseases [232, 233]. Citrullination is the process by which peptidyl arginine deiminases (PADIs) catalyze the deamination of arginine to citrulline. Since its discovery, it has been established that protein citrullination plays a role in bone diseases, particularly rheumatoid arthritis (RA).

In ROS-induced senescent osteoblasts, down-regulated PADI2 contributes to the activation and nuclear translocation of NF-κB p65. p65 facilitates the release of SASP, including chemokine ligand (CCL) 2, CCL5, and CCL7, and causes cellular senescence in osteoblasts [22]. Mice in which *Padi2* was specifically deleted in osteoblasts were bred to confirm the link between PADI2 and osteoblast differentiation. In fact, the mice exhibited decreased bone mass due to impaired bone formation and enhanced osteoclast activity [22]. Another investigation revealed that RUNX2 has ten arginine sites that could be citrullinated. Similar to acetylation and lactylation, the citrullination of R381 (the 381^st^ arginine) promotes RUNX2 stability and osteogenic development by preventing RUNX2 degradation via the ubiquitin-protease pathway [234].

As members of hematopoietic lineage cells, dendritic cells (DCs) can differentiate into osteoclasts under the induction of RANKL and M-CSF. During osteoclast differentiation, researchers found elevated PADI2 and PADI4 expression and increased citrullinated proteins [235]. Among the proteins, citrullinated actin and vimentin facilitate osteoclast differentiation and aggravate arthritis. Additionally, higher levels of citrullinated vimentin are exhibited in the gingival crevicular fluids and alveolar bone surfaces of mice with periodontitis. Moreover, the addition of citrullinated vimentin promotes osteoclast activity and the formation of resorption pits via protein kinase Cδ (PKCδ)/ERK signaling [236]. While native fibrinogen exhibits a bone-protective function, citrullinated fibrinogen contributes to the production of genes and proteins related to osteoclast differentiation and bone resorption [237].

### 5.7 PTMs that couple bone metabolism

Though extensive research has been performed on PTMs, there are still several discrepancies that require further investigation to be comprehensively explained. As histone modifications with metabolites as substrates usually facilitate gene expression, it is difficult to definitively conclude their specific roles in bone metabolism. This might be due to the complexity of intracellular signaling and intercellular communication among the participating cells in bone metabolism. For example, histone acetylation has been shown to promote osteogenesis or inhibit it in different studies. In fact, various downstream genes might explain this discrepancy. Both RUNX2 and PPARγ can be upregulated by H3K27ac, though they have been shown to oppositely regulate differentiation pathways of BMSCs [112, 116]. Additionally, in BMSCs, p300 induces H3K27ac to facilitate RUNX2 expression, while p300 also contributes to NFATc1 expression in osteoclasts [111, 171]. However, p300 might not be upregulated in osteoblasts and osteoclasts simultaneously. Bone resorption mediated by osteoclasts and bone formation mediated by osteoblasts are coordinated spatiotemporally during bone remodeling. Thus, the sequential activation of cell differentiation into osteoclasts or osteoblasts could not be easily simulated when cells are cultured *in vitro*. The results obtained from *in vitro* studies could only reflect potential changes in cells under an induction microenvironment, which differs from *in vivo* conditions. We hypothesize that histone acetylation might facilitate osteoclast differentiation during the initial phase of bone resorption. Subsequently, residual acetyl CoA produced by activated osteoclasts might serve as the substrate of the following histone acetylation in osteoblasts to facilitate osteogenesis in the reversal stage. The different PTMs on non-histones also result in conflicting alterations of protein functions. This might be due to differential conformational changes resulting from the binding or unbinding of such metabolites at various amino acid residues [238]. Therefore, the same PTMs on different sites of a specific protein could lead to opposite outcomes. Clearly, further investigations on the entire bone remodeling process and PTMs of histones and non-histones are necessary to fully understand their comprehensive mechanisms.

In order to target PTMs to solve osteoporosis, it is necessary to focus on PTMs with the opposite influences on osteogenesis and osteoclastogenesis. Evidently, as members of sirtuins, SIRT1 and SIRT6 protect bone by enhancing bone formation and inhibiting bone resorption. For instance, SIRT1 induced deacetylation of H3K9ac in the gene promoter of *Sost*, which encodes an inhibitory protein of Wnt signaling [118]. Clearly, SIRT1 contributes to osteogenesis. Additionally, SIRT1 inhibits osteoclast differentiation through FOXO1 deacetylation, and deacetylated FOXO1 impairs OXPHOS and the production of ATP in osteoclasts [184]. Therefore, the activation of SIRT1 might successfully rescue bone mass by inhibiting bone resorption and enhancing bone formation simultaneously. In contrast, HDAC1 and HDAC3 exert opposite effects on bone metabolism compared to SIRT1. Therefore, the specific inhibitors of HDAC1 and HDAC3 could enhance bone mass by promoting osteogenesis and suppressing osteoclastogenesis. Furthermore, the acetylation at different sites of a specific protein can have opposing functional effects. Point mutation could be utilized to alter the amino acid type at specific sites to control PTMs. In general, the PTMs on histones lead to enhanced chromatin accessibility and increased gene expression. It is difficult to target a specific histone modification site since the downstream genes are too abundant. Therefore, it is more probable to target PTMs of non-histones to alleviate osteoporosis. FOXO1 and p65 might be key candidates for further investigation, as they have been shown to regulate osteogenesis and osteoclastogenesis in opposing ways via distinct acetylated sites.

### 5.8 Other metabolites that affect bone metabolism

Apart from the PTMs discussed above, other metabolites could regulate cellular processes and biological activities through PTMs, such as protein propionylation or β-hydroxybutyrylation. However, though the metabolites could regulate bone metabolism, it remains uncertain whether these modifications influence osteogenesis or bone resorption. For example, unsaturated fatty acids have been shown to promote bone regeneration and reduce the incidence of osteoporosis [239, 240]. By reducing NFATc1 expression in osteoclasts, β-hydroxybutyric acid (BHB) inhibits osteoclast differentiation and rescues the osteoporosis phenotype [241]. Elevated 2-hydroxyisobutyrylation is observed in induced pluripotent stem cells (iPSCs) extracted from the urine of patients with osteosclerosis compared to those from healthy controls, suggesting that 2-hydroxyisobutyrylation might influence osteogenesis [242]. Additionally, arginine and methionine are necessary for osteoblast differentiation and bone homeostasis, as they have been shown to facilitate osteogenesis and angiogenesis but inhibit adipogenesis both *in vitro* and *in vivo* [243, 244]. Therefore, we anticipate that additional research on the relationship between metabolite-mediated PTMs and bone homeostasis is needed. It will contribute to a better understanding of the bone system and provide hypotheses for potential osteoporosis treatments.

## 6. Preparations for the clinical transduction of PTMs

To address osteoporosis through PTMs, drugs could be designed to target metabolites or regulatory enzymes. Due to the interactions among metabolic pathways, it is difficult to utilize the metabolites to treat osteoporosis. For example, lactate, a glycolysis end product, could induce OXPHOS to generate energy [245]. Thus, the final PTM transitions after following lactate supplement are unpredictable. Targeting regulatory enzymes such as sirtuins or HDACs appears more feasible. However, PTMs are involved in pathologies of systemic diseases such as cancer, chronic kidney disease, and other metabolic disorders, beyond their role in bone metabolism [246-248]. Therefore, drug safety profiles must be rigorously validated prior to clinical use to minimize off-target effects. Currently, reliable methods to assess the levels of PTMs in osteoporotic patients require development. These methods could establish standardized criteria and provide guidance for the clinical treatment protocols. Due to the incomplete understanding of PTM mechanisms in bone metabolism, developing effective therapies remains challenging. These challenges must be addressed prior to the clinical trials and applications.

## 7. Conclusion and Perspective

Summarized in this review, healthy bone turnover depends on the coordinated metabolism of glucose, lipids, and amino acids. Metabolic rewiring occurs during osteoblast and osteoclast differentiation and leads to altered metabolite profiles. Metabolites could be substrates for PTMs. Thus, through PTMs, they could regulate cell fates, such as cell proliferation, differentiation, and apoptosis. Both humans and animals with osteoporosis exhibit altered metabolism and metabolites, as well as distinct PTM expression patterns that may contribute to bone fragility.

Decreased histone acetylation in MSCs during aging impairs osteogenesis, as histone acetylation induces chromatin relaxation and enhances osteogenic gene expression. However, sirtuins, recognized as longevity proteins, promote histone deacetylation yet alleviate osteoporosis. The discrepancy might be caused by the various downstream genes that are regulated by the same histone acetylation site. In osteoclasts, RANKL induces the assembly of the NCoR/HDAC3/PGC1β complex, which reverses the deacetylation activity of HDAC3 and recruits p300. Thus, enhanced H3K27ac induces the expression of osteoclastic genes and contributes to osteoclast differentiation. Similar to histone acetylation, histone lactylation promotes osteogenesis. Meanwhile, the two PTMs share common writers and erasers. By occupying ubiquitination sites, non-histone acetylation and lactylation increase protein stability and prevent degradation via ubiquitination. Palmitoylation of the receptors is necessary for their proper positioning and structural integrity. These are essential for their binding to the specific ligands. In general, protein palmitoylation contributes to bone mass, since the *Zdhhc13*-deficient mice exhibit severe osteoporosis. The citrullinated vimentin and fibronectin stimulate osteoclast differentiation and bone resorption in periodontitis, suggesting that neutralizing antibodies might be an effective treatment for excessive citrullination in periodontitis.

In fact, the comprehensive metabolism in the bone microenvironment has not been entirely verified due to its complexity. Given the complexity of metabolism, the role of PTMs with metabolites as substrates in bone metabolism requires further investigation. Additionally, even though extensive studies on PTMs have been conducted, key questions remain unresolved. For example, since both H3K18la and H3K18ac can facilitate osteoblast differentiation of MSCs, which modification predominantly drives this process? Since several PTMs share common writers, readers and erasers, how do they coordinate harmoniously? In addition to acetylation, can other PTMs be transported via EVs or biological materials to mediate bone turnover? Therefore, further investigations are required to identify new PTMs with diverse metabolites as substrates and elucidate the complicated regulatory networks. We believe that a comprehensive understanding of the mechanism of PTMs in osteoporosis will advance clinical treatment.
